# Gout and risk of dementia, Alzheimer's disease or vascular dementia: a meta-epidemiology study

**DOI:** 10.3389/fnagi.2023.1051809

**Published:** 2023-04-26

**Authors:** Xuanlin Li, Lin Huang, Yujun Tang, Xuanming Hu, Chengping Wen

**Affiliations:** College of Basic Medical Science, Zhejiang Chinese Medical University, Hangzhou, China

**Keywords:** gout, dementia, Alzheimer's disease, vascular dementia, meta-analysis

## Abstract

**Objectives:**

The association between gout and dementia, Alzheimer's disease (AD), or vascular dementia (VD) is not fully understood. The aim of this meta-analysis was to evaluate the risk of all-cause dementia, AD, and VD in gout patients with or without medication.

**Methods:**

Data sources were PubMed, Embase, the Cochrane Library, and reference lists of included studies. This meta-analysis included cohort studies assessing whether the risk of all-cause dementia, AD, and VD was associated with gout. The risk of bias was assessed using the Newcastle-Ottawa Quality Assessment Scale (NOS). The Grading of Recommendations Assessment, Development, and Evaluation (GRADE) system was used to access the overall certainty of evidence. Risk ratios (*RR*) with 95% confidence intervals (*CI*) were pooled using a random-effects model, and publication bias was assessed with funnel plots and Egger's test.

**Results:**

A total of six cohort studies involving 2,349,605 individuals were included in this meta-analysis, which were published between 2015 and 2022. The pooling analysis shows that the risk of all-cause dementia was decreased in gout patients [*RR* = 0.67, 95% *CI* (0.51, 0.89), *I*^2^ = 99%, *P* = 0.005, very low quality], especially in gout patients with medication [*RR* = 0.50, 95% *CI* (0.31, 0.79), *I*^2^ = 93%, *P* = 0.003, low quality]. The risk of AD [*RR* = 0.70, 95% *CI* (0.63, 0.79), *I*^2^ = 57.2%, *P* = 0.000, very low quality] and VD [*RR* = 0.68, 95% *CI* (0.49, 0.95), *I*^2^ = 91.2%, *P* = 0.025, very low quality] was also decreased in gout patients. Despite the large heterogeneity, the sensitivity analysis indicated that the results were robust, and there was little evidence of publication bias.

**Conclusion:**

The risk of all-cause dementia, AD, and VD is decreased in gout patients, but the quality of evidence is generally low. More studies are still needed to validate and explore the mechanisms of this association.

**Systematic review registration:**

https://www.crd.york.ac.uk/prospero/#recordDetails, identifier: CRD42022353312.

## 1. Introduction

Dementia is one of the most common diseases with high morbidity, mortality, and reduced quality of life, especially in the elderly population (Frederiksen et al., 2020). Notably, the number of dementia patients or related cognitive impairment is expected to increase to 115 million by 2050 as the world enters an aging era (Bai et al., 2022). A study that estimated the prevalence of young-onset dementia using a large study population and multiple national health datasets showed a trend toward younger onset of dementia (Ryan et al., 2022). Currently, approximately two-thirds of dementia patients live in low- and middle-income countries, and the average total national expenditure on dementia is accounting for 0.45% of the national gross domestic product (GDP) in these countries. The cost of dementia care increases with the severity of dementia and the number of comorbidities, with total national dementia costs estimated to range from $1.04 million per year in Vanuatu to $150 billion per year in China (Mattap et al., 2022). Therefore, it is important to actively and effectively prevent the onset and progression of dementia and its related diseases such as Alzheimer's disease (AD) and vascular dementia (VD).

Gout is a debilitating disease with long-term complications, including joint damage and urate deposit stones (Neilson et al., 2022). It is estimated that there are ~41.2 million prevalent gout cases globally, with 7.4 million gout cases annually and nearly 1.3 million disabled gout patients in 2017 (Safiri et al., 2020). Gout is the most common inflammatory arthritis in adults. Recently, the dysregulation of the immune system has received much attention in the pathogenesis of neurodegenerative diseases and is even considered as important as the classical hypothesis of pathological protein aggregation. A prospective cohort study of 375,894 individuals showed that immune-mediated disorders were significantly associated with an increased risk of dementia (Zhang et al., 2022). In addition, several observational studies have reported that gout and hyperuricemia might increase the risk of dementia, although the conclusions of different studies are inconsistent (Hong et al., 2015; Lu et al., 2016; Singh and Cleveland, 2018; Chuang et al., 2021; Mikhailichenko et al., 2021; Min et al., 2021; Dehlin et al., 2022; Kim et al., 2022). One recent meta-analysis on a similar topic has been published. This meta-analysis by Pan et al. included four cohorts and found that gout and hyperuricemia did not increase the risk of dementia but might decrease the risk of AD (Pan et al., 2021). However, this pooled analysis included patients with high uric acid who were not clearly diagnosed with gout (Latourte et al., 2018), and there may have been patient selection bias. Furthermore, the inclusion of gout patients with or without medication intervention during the follow-up period was not properly differentiated. Recently, several large samples and well-designed cohort studies involving new evidence about the risk of all-cause dementia have been published (Chuang et al., 2021; Min et al., 2021; Kim et al., 2022). Because of the importance of the issue, the limitations of the previous review, and the availability of new evidence, we conducted a systematic review and meta-analysis to evaluate the association between gout alone and the risk of all-cause dementia.

## 2. Methods

This meta-analysis is reported according to the updated guidance and exemplars for reporting systematic reviews (PRISMA 2020 statement; Page et al., 2021), and our protocol was registered on PROSPERO (CRD42022353312).

### 2.1. Data sources

PubMed, EMBASE, and Cochrane Library were searched without any restrictions from inception to 16 August 2022. The subject terms (Emtree in Embase, MeSH in PubMed) and corresponding keywords were used. Search terms included those related to dementia, Alzheimer's disease, and gout and its variants. The reference lists of retrieved studies and previous meta-analyses were also checked to identify other studies that might be eligible for inclusion. The full search strategy for these databases is provided in Supplementary Tables 1–3.

### 2.2. Study selection

The retrieved initial records were imported into NoteExpress reference management software, and duplicate records were removed. In total, two authors (XL Li and XM Hu) independently reviewed titles and abstracts to exclude irrelevant records and then classified the remaining records according to inclusion, exclusion, or uncertainty. For records that were uncertain, the full text was read to ensure eligibility for inclusion. Any disputes were resolved through group discussion.

### 2.3. Eligibility criteria

Studies were considered eligible if they met the following criteria: (a) patients: dementia, Alzheimer's disease (AD), or vascular dementia (VD); (b) exposure: a definitive diagnosis of gout, not just hyperuricemia, which is only a biochemical precursor to gout; (c) comparator: healthy people or non-gout sufferers; (d) outcomes: a presentation of quantitative point estimates [hazard ratios (HRs), relative risks (RRs), or odds ratios (ORs)] and a variance of the estimates of the association between gout and the risk of dementia, AD, or VD and also a description of adjustment for potential confounders (Shang et al., 2021; Cao et al., 2022); and (e) type of study: cohort study, either prospective or retrospective.

The exclusion criteria were as follows: (a) conference abstracts or letters to editors; (b) duplicate publication; (c) patients diagnosed with increased uric acid or hyperuricemia; and (d) incomplete data or no interested outcome.

### 2.4. Data extraction

We designed a data extraction form in Excel (Microsoft Corporation, USA). In total, two authors (XL Li and YJ Tang) independently extracted information from eligible cohorts. The following data were obtained from each study: first author, time of publication, country, number of events, number of exposures, confounders, and so on. The extracted data were cross-checked, and disagreements were resolved through discussion.

### 2.5. Study quality

The Newcastle-Ottawa Quality Assessment Scale (NOS; Available from: http://www.ohri.ca/programs/clinical_epidemiology/oxford.asp) was used to evaluate the quality of the included studies from three aspects: selection, comparison, and outcomes. Scores for cohort and case–control studies ranged from 0 to 9 stars, with higher stars indicating higher study quality. NOS stars ≥7, 4–6, and 0–3 were of high, moderate, and low quality, respectively.

### 2.6. Evidence certainty

The Grading of Recommendations Assessment, Development, and Evaluation (GRADE) system (Balshem et al., 2011) was used to access the overall certainty of evidence. By the GRADE system, the certainty of evidence derived from cohort studies receives an initial grade of low quality. The quality of evidence from cohort studies can be improved at larger effect sizes (*RR* ≥ 2 or ≤ 0.5), dose–response gradients, or attenuation by plausible confounding after excluding various factors that could lead to downgrading (Guyatt et al., 2011). Finally, the evidence of outcomes can be graded as high, moderate, low, or very low.

### 2.7. Data synthesis

The Stata software (version 14) was used to conduct the data analysis. We assessed heterogeneity using the chi-square test and *I*^2^ value, and *P* < 0.1 or *I*^2^ > 50% indicated that heterogeneity was great, and thus, the random-effects model was adopted (Higgins et al., 2003). Otherwise, a fixed-effect model was employed according to a previous high-quality meta-analysis (Lei et al., 2022; Qu et al., 2022). The sensitivity analysis was performed to verify the robustness of the overall results and to explore sources of heterogeneity. The subgroup analysis was performed according to whether patients with gout received medication intervention. Finally, funnel plots, Begg's test, and Egger's regression test were used to detect publication bias (Egger et al., 1997).

## 3. Results

### 3.1. Study selection

**Figure 1:**
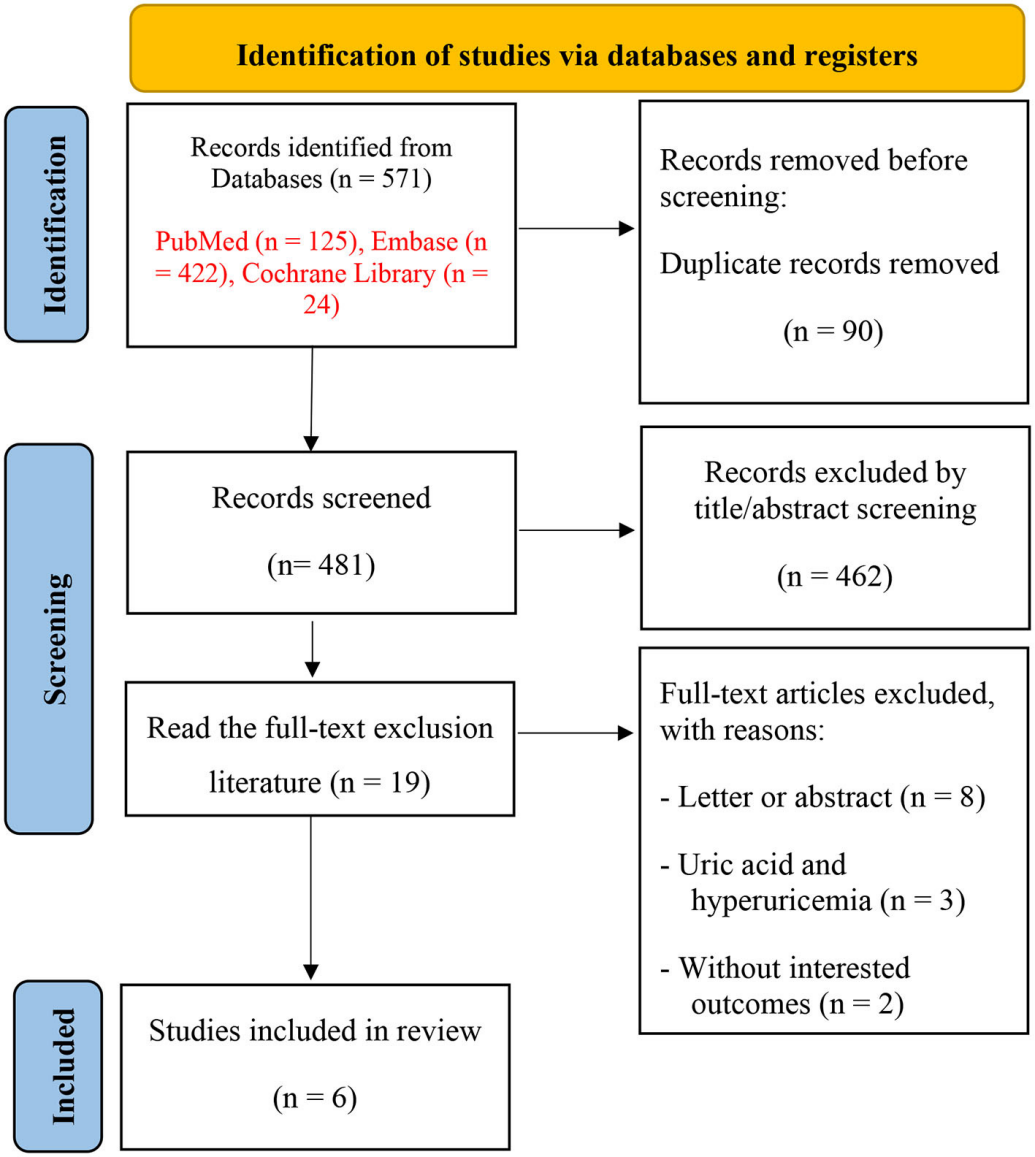
Literature screening flowchart.

Our search yielded 571 related records, and 90 were excluded due to duplication. Of these, 462 were eliminated by screening titles and abstracts for being irrelevant to our topic. The remaining 19 studies were scrutinized for further assessment. Finally, a total of six cohort studies (Hong et al., 2015; Lu et al., 2016; Singh and Cleveland, 2018; Chuang et al., 2021; Min et al., 2021; Kim et al., 2022) were included in the meta-analysis. The study selection process is shown in Figure 1.

### 3.2. Study characteristics

A total of six cohort studies (Hong et al., 2015; Lu et al., 2016; Singh and Cleveland, 2018; Chuang et al., 2021; Min et al., 2021; Kim et al., 2022) involving 2,349,605 individuals (415,653 with gout and 19,497 with dementia events) were included. The studies were published between 2015 and 2022, with the sample size ranging from 28,624 to 1,712,821, and the follow-up years were from 2.3 to 11 years. In total, two studies were conducted in Korea (Min et al., 2021; Kim et al., 2022), two in China (Hong et al., 2015; Chuang et al., 2021), and another two were performed in the United States (Singh and Cleveland, 2018) and the United Kingdom (Lu et al., 2016), respectively. In the original study included, the diagnosis of dementia and gout was mostly classified and diagnosed according to the standard of “International Classification of Diseases (ICD).” The adjusted confounders are slightly different in included studies, and age, sex, and body mass index (BMI) were the most common adjusted confounders. The characteristics of six cohort studies are summarized in Table 1.

**Table 1:** Characteristics of studies included in the review.

**References**	**Country**	**Study type**	**Sample size**	**No. of outcome**	**Follow up period**	**Baseline age (years)**	**Diagnosis of gout**	**Diagnosis of dementia**	**Confounders adjusted**
Kim et al. (2022)	Korea	Retrospective cohort	Total: 30,312, gout: 5,052, no gout: 25,260	Dementia: 639	2002–2013	56.87 ± 13.78	ICD-10, M10	ICD-10, F00, and F01	Age, sex, household income, diabetes, hypertension, dyslipidemia, stroke/transient ischemic attack, and depression.
Min et al. (2021)	Korea	Retrospective cohort	Total: 136,308, gout: 22,718, no gout: 113,590	Dementia: 2,557	2002–2013	72.29 ± 6.10	ICD-10, M10	ICD-10 codes “F00,” “F01,” “F02,” and “F03”	Age, female sex, cardiovascular disease, diabetes mellitus, hypertension, dyslipidemia, Parkinson's disease, depression, traumatic brain injuries, average income
Singh and Cleveland (2018)	USA	observational cohort	Total: 1712821, gout: 296648, no gout:1416173	Dementia: 5310	2006 ~ 2012	75.2 ± 7.5	ICD-9-CM code of 274.xx	ICD-9-CM codes for 290.xx, 294.1x, or 331.2	Age, sex, BMI, entry-time
Lu et al. (2016)	UK	Retrospective cohort	Total: 298,029, gout: 59,224, no gout: 238,805	Dementia: 2,251	1995–2021	65.3 ± 12.2	READ codes (O'Neil et al., 1995)	ICD9-CM code 290.4	Age, sex, BMI, Diuretics, other CV drugs, cardiovascular comorbidities, smoking, alcohol, social-economic deprivation index
Hong et al. (2015)	China	Retrospective cohort	Total: 143,511, gout: 28,769, no gout: 114,742	Dementia: 7,119	2002–2008	63.5 ± 9.7	ICD9-CM code 274	ICD9-CM code 331.0 and 290.0–290.4	Age, sex, diabetes mellitus, hypertension, hyperlipidemia, heart failure, coronary artery disease, COPD, asthma, malignancy, arrhythmia, Parkinson's disease
Chuang et al. (2021)	China	Retrospective cohort	Total: 28,624, gout: 3,242, no gout: 25,382	Dementia: 1,621	2002–2008	76.9 ± 7.1	ICD-9-CM code 274	ICD-9-CM codes: 290.0–290.4, 294.1, and 331.0–331.2	Allopurinol, benzbromarone, sulfinpyrazone, probenecid, hypertension, hyperlipidemia, CLD, CKD, DM, COPD, AD, CVD, stroke, warfarin, and statin

ICD, International Classification of Diseases.

### 3.3. Quality assessment

All six cohorts (Hong et al., 2015; Lu et al., 2016; Singh and Cleveland, 2018; Chuang et al., 2021; Min et al., 2021; Kim et al., 2022) have a score of ≥7, which indicates that the studies included in this meta-analysis are of high quality. Details of the risk of bias are summarized in Table 2.

**Table 2:** Newcastle-Ottawa quality of cohort studies.

**References**	**Selection**	**Comparability**	**Outcome**	**Overall quality score**
Kim et al. (2022)	****	**	**	8
Min et al. (2021)	***	**	**	7
Singh and Cleveland (2018)	****	**	**	8
Lu et al. (2016)	****	**	**	8
Hong et al. (2015)	****	**	**	8
Chuang et al. (2021)	***	**	**	7

### 3.4. Gout and risk of dementia

**Figure 2:**
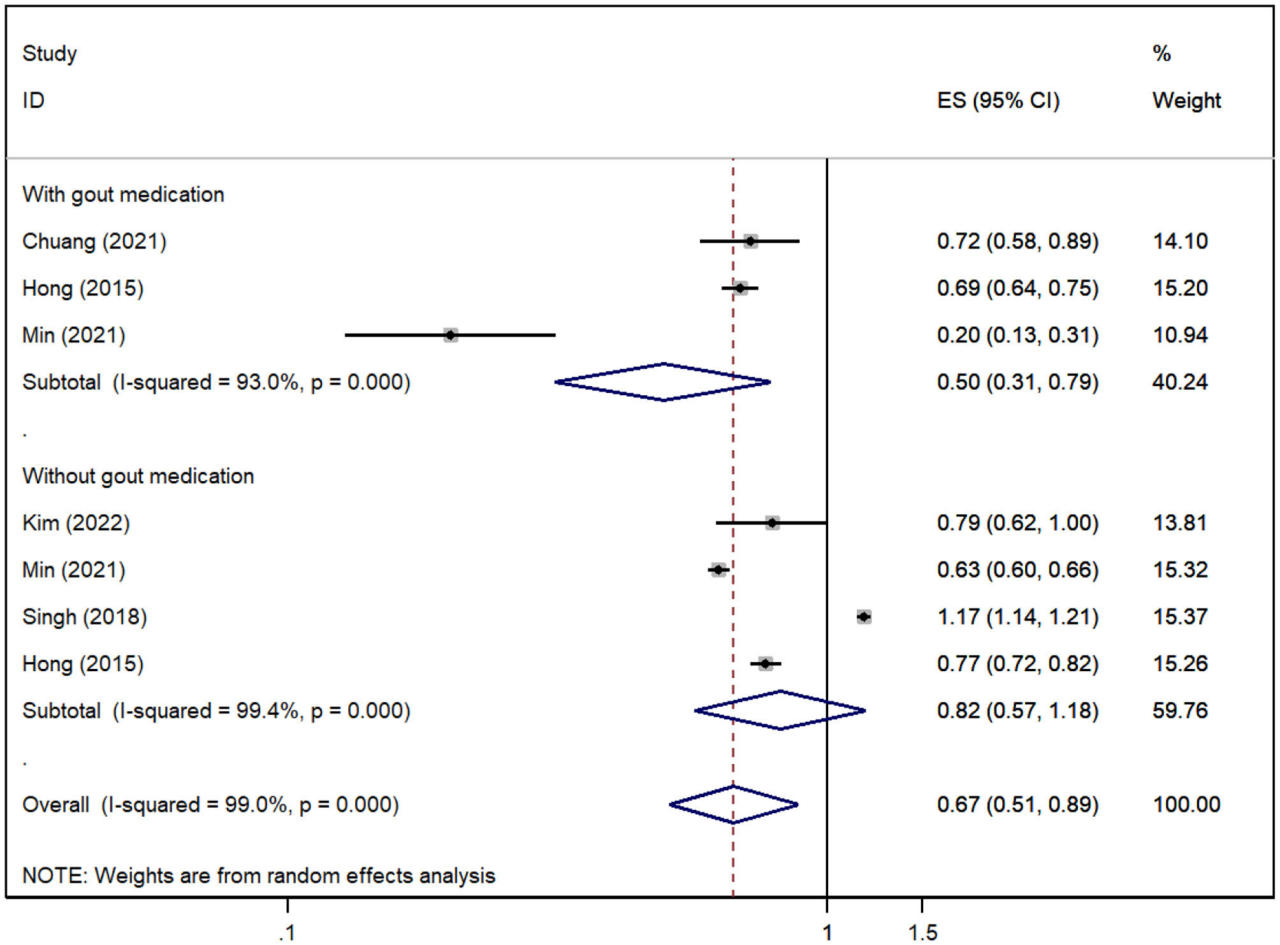
Forest plot for the risk of all-cause dementia in gout patients. ES, effect size.

A total of five cohorts (Hong et al., 2015; Singh and Cleveland, 2018; Chuang et al., 2021; Min et al., 2021; Kim et al., 2022) reported the risk of all-cause dementia in gout patients, and the pooled analysis suggested that the risk of all-cause dementia was decreased in gout patients [*RR* = 0.67, 95% *CI* (0.51, 0.89), *I*^2^ = 99%, *P* = 0.005; Figure 2]. Due to significant heterogeneity, we performed sensitivity analysis by removing each study to explore the source of heterogeneity, which suggested that the results were stable after excluding any one study, suggesting that the results of our meta-analysis were robust (Supplementary Figure 1). However, the results for subgroups stratified according to whether they had medication interventions for gout were inconsistent. The risk of all-cause dementia was significantly reduced when gout patients were treated with medication (Hong et al., 2015; Chuang et al., 2021; Min et al., 2021) [*RR* = 0.50, 95% *CI* (0.31, 0.79), *I*^2^ = 93%, *P* = 0.003; Figure 2], but no association was found when no medication was administered in gout patients (Hong et al., 2015; Singh and Cleveland, 2018; Min et al., 2021; Kim et al., 2022) [*RR* = 0.82, 95% *CI* (0.57, 1.18), *I*^2^ = 99.4%, *P* = 0.285; Figure 2].

### 3.5. Gout and risk of AD

**Figure 3:**
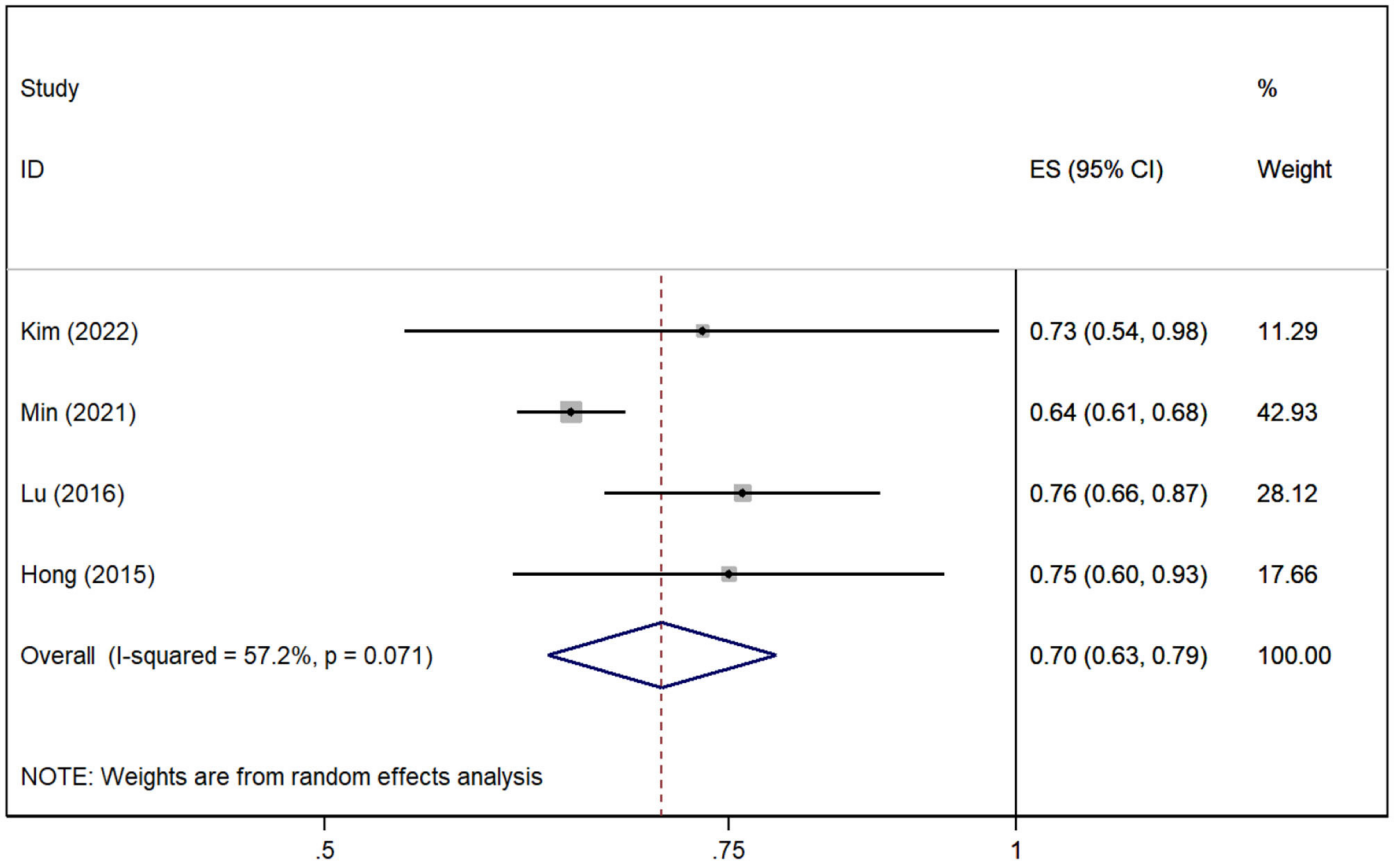
Forest plot for the risk of AD in gout patients. ES, effect size.

A total of four cohort studies (Hong et al., 2015; Lu et al., 2016; Min et al., 2021; Kim et al., 2022) assessed the association between gout without medication and the risk of AD. The pooled analysis showed that gout patients are associated with decreased risk of AD [*RR* = 0.70, 95% *CI* (0.63, 0.79), *I*^2^ = 57.2%, *P* = 0.000; Figure 3]. The sensitivity analysis showed that none of the studies reversed the pooled effect, which means that the results of the risk of AD in gout are robust (Supplementary Figure 2).

### 3.6. Gout and risk of VD

**Figure 4:**
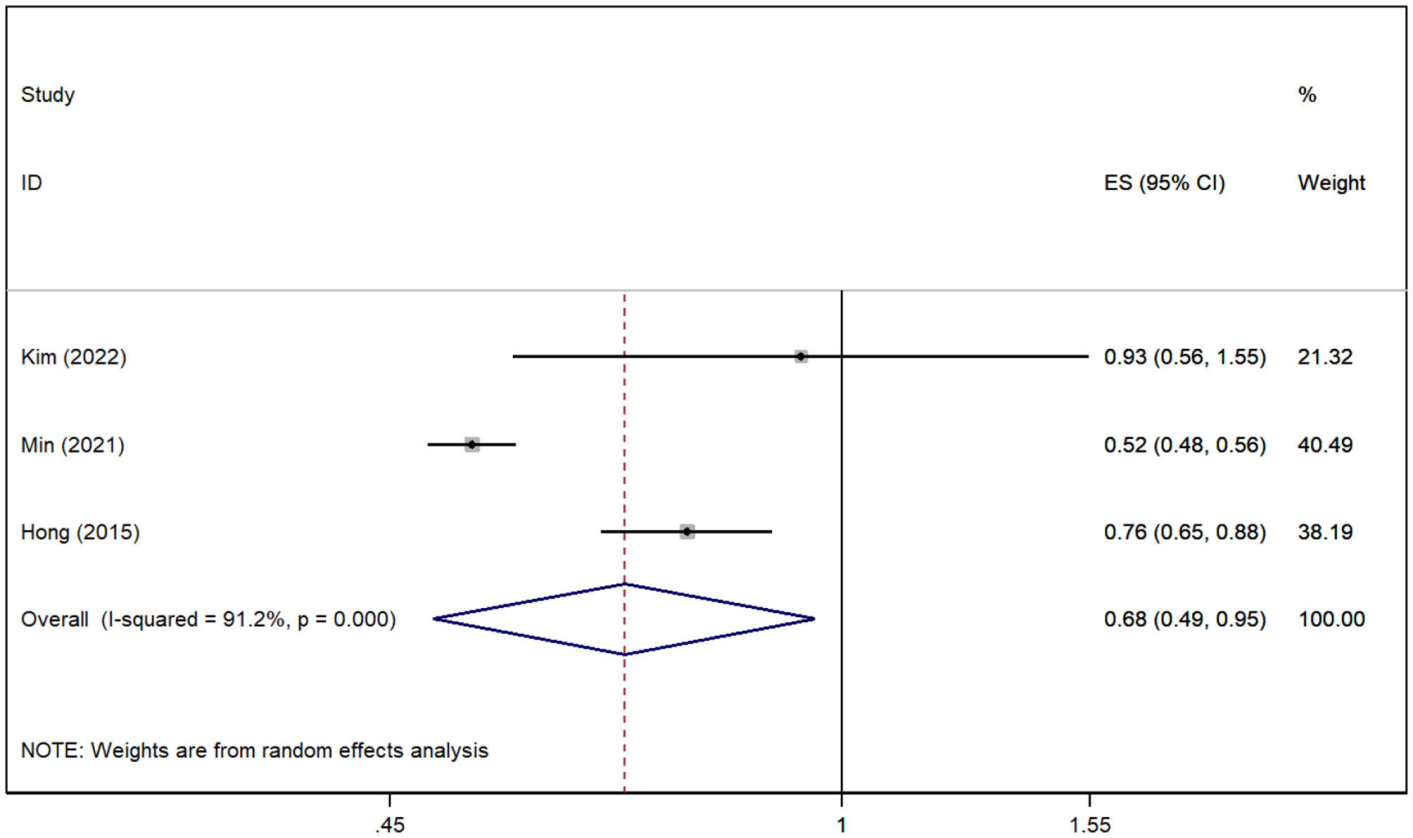
Forest plot for the risk of VD in gout patients. ES, effect size.

In total, three studies (Hong et al., 2015; Min et al., 2021; Kim et al., 2022) revealed the relationship between gout and the risk of VD. The pooled analysis showed that the risk of VD was reduced in patients with gout [*RR* = 0.68, 95% *CI* (0.49, 0.95), *I*^2^ = 91.2%, *P* = 0.025; Figure 4]. Although there was large heterogeneity, the results of sensitivity analysis support the robustness of this result.

### 3.7. Evidence certainty

The GRADE level of evidence is very low for the risk of dementia, risk of AD, and risk of VD in gout patients without medication and is low for the risk of dementia in gout patients with medication. GRADE evidence certainty for the outcomes is shown in Table 3.

**Table 3:** GRADE certainty of evidence.

**Outcome**	**Exposure**	**Study numbers**	**GRADE**					**Evidence quality**

			**Risk of bias**	**Inconsistency**	**Indirect ness**	**Imprecision**	**Publication bias**	
Dementia	Gout with medication	3	0	−1^a^	0	+1^b^	0	Low
Dementia	Gout	5	0	−1^a^	0	0	0	Very low
AD	Gout without medication	4	0	−1^a^	0	0	0	Very low
VD	Gout without medication	3	0	−1^a^	0	0	0	Very low

AD, Alzheimer's disease; VD, vascular dementia.

^a^High heterogeneity.

^b^Large effect sizes.

### 3.8. Publication bias

**Figure 5:**
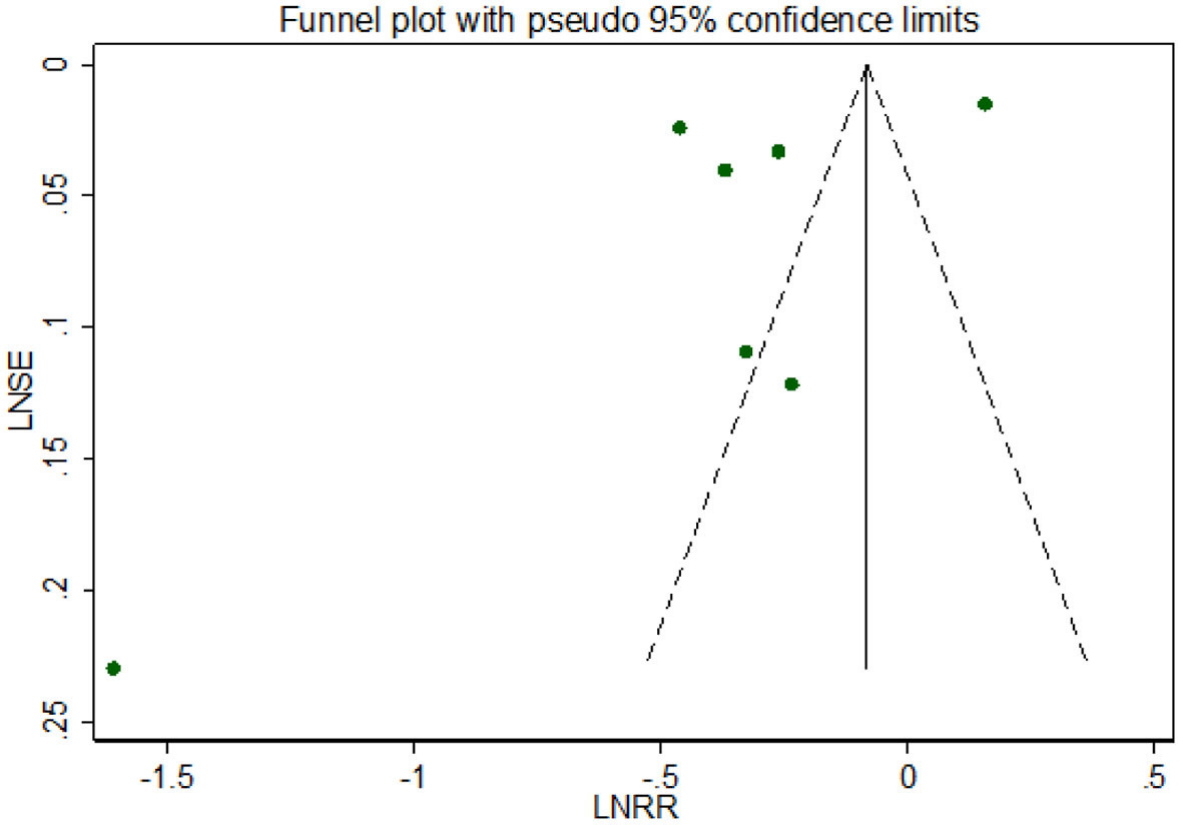
Funnel plot for all-cause dementia in gout patients.

Visual inspection suggested that the funnel plot for all-cause dementia in gout patients was symmetrical but in formal statistical tests, including Egger's test (*p* = 0.197) and Begg's test (*p* = 0.881), no publication bias was shown. The funnel plot is shown in Figure 5.

## 4. Discussion

### 4.1. Principal findings

Our meta-analysis involving 2,349,605 individuals found a reduced risk of all-cause dementia in patients with gout, particularly in those with a history of medication therapy. Meanwhile, gout seems to be a protective factor for AD and VD although the quality of evidence is low or very low.

### 4.2. Comparison with previous studies

A total of one meta-analysis by Pan et al. reported that gout and hyperuricemia did not increase the risk of dementia but might decrease the risk of AD (Pan et al., 2021). However, in the four included cohort studies, one study (Latourte et al., 2018) only investigated the risk of dementia and AD in hyperuricemia patients. We all know that hyperuricemia is a biochemical precursor to gout, and nearly two-thirds of patients will not be diagnosed with gout (Clarson et al., 2015). Therefore, this study (Latourte et al., 2018) was excluded from our meta-analysis, thereby reducing patient selection bias and confounding. Moreover, the meta-analysis of Pan et al. (2021) did not distinguish between the special case of adherence to medication in the cohort follow-up, which may introduce confounding in the intervention. Thus, our meta-analysis distinguished between the two types of gout patients and dementia-onset outcomes based on the presence or absence of medication. In comparison, our meta-analysis found that the risk of all-cause dementia was reduced in gout patients, and this phenomenon was more pronounced in gout patients who were treated with medication. This may be due to our inclusion of several large samples of high-quality cohort studies while focusing more on patients with gout and controlling for other confounders. Notably, similar to the results of Pan et al. (2021), we found that the risk of AD was also reduced in gout patients without medication. In addition, we further evaluated the association of gout with the risk of VD, and the risk of VD was also reduced in gout patients, which has not been reported in a previous meta-analysis (Pan et al., 2021).

### 4.3. Interpretation of findings

To date, whether gout or hyperuricemia contributes to the risk of all-cause dementia has been controversial. There are several main postulated mechanisms of all-cause dementia caused by gout or hyperuricemia, but the more focused ones are chronic inflammation and oxidative stress mechanisms (Campbell et al., 1997; Lin and Beal, 2006; Bhat et al., 2015; Flores-Aguilar et al., 2021; Jurcau, 2021). Chronic inflammation caused by gout or hyperuricemia increases inflammatory cytokine levels in the brain, leading to neurodegenerative lesions. Meanwhile, high inflammatory cytokines damage endothelial cells and activate inflammatory cells, causing oxidative stress and promoting the development of atherosclerosis, a recognized risk factor for dementia (Lin and Beal, 2006; Bhat et al., 2015; Jurcau, 2021). However, based on the result of our meta-analysis, the risk of all-cause dementia, AD, and VD was low in gout patients, especially in patients with medication management. The explanatory mechanism for these findings remains to be explored. An important mechanism may be the pro- and antioxidant effects of uric acid. Uric acid is an important circulating antioxidant and free radical scavenger, which can scavenge peroxynitrite and hydroxyl radicals and reduce oxidative stress, thereby exerting neuroprotective effects (Kanellis and Kang, 2005; Lee et al., 2022). In addition, uric acid may preserve mitochondrial function and inhibit the accumulation of oxygen-free radicals, and mitochondrial dysfunction is considered to be one of the pathogeneses of AD (Yu et al., 1998; Ye et al., 2016). In clinical observations, Euser et al. found that high serum urate levels were associated with a reduced risk of dementia and further improved cognitive function (Euser et al., 2009), and Scheepers et al. found that higher serum uric acid may be protective against dementia including AD and VD (Scheepers et al., 2019). These are consistent with the findings of our meta-analysis. In addition, the gout patients who adhered to drug treatment in this study had a lower risk of dementia, which may be related to some uric acid-lowering drugs to antagonize oxidative stress and improve vascular endothelial cell function (Kryscio et al., 2017). The results of our meta-analysis give great insight to both clinicians and gout patients: first, to insist on proper management of gout, and second, to reduce the psychological burden by recognizing the uncertainty of the dangers of gout in terms of causing dementia or AD.

### 4.4. Strengths and limitations

The main advantage of our meta-analysis is the application of the GRADE system for grading the quality of evidence, which seems to be relatively rare in previous meta-analyses based on observational studies. Moreover, it is registered in PROSPERO and reported according to the updated PRISMA checklist, promoting transparency of the process and reliability of the results. However, this meta-analysis inevitably has several potential shortcomings. First, although we require a clear diagnosis of gout and a subgroup analysis based on medication taken, there are some clinical heterogeneities in the severity, disease status, and duration of disease in these gout patients which may be inconsistent. Second, there is a certain degree of statistical heterogeneity in the larger *I*^2^ of our meta-analysis. Although the sensitivity analysis indicates that the results are relatively robust, it inevitably affects the reliability of the results. These also contributed to the overall low quality of evidence. Finally, although we conducted a comprehensive literature search and excluded conference abstracts (incomplete information could lead to a biased assessment of methodological quality), it was difficult to exclude studies that might have been missed.

## 5. Conclusion

Our updated meta-analysis found that the risk of all-cause dementia, AD, and VD was reduced in patients with gout, but there was a large heterogeneity, and the mechanism was not clear. More studies are needed to investigate the true relationship between gout and dementia, AD, and VD in future.

## Data availability statement

The original contributions presented in the study are included in the article/Supplementary material, further inquiries can be directed to the corresponding author.

## Author contributions

XL: acquisition of data, analysis and interpretation of data, drafting of the article, and approval of the final version to be submitted. XH and YT: acquisition of data, analysis and interpretation of data, and final approval of the version to be submitted. LH and CW: conception and design of the study, revision of the manuscript, and final approval of the version to be submitted. All authors contributed to the article and approved the submitted version.
