# Supplementary material for: Gout and risk of dementia, Alzheimer's disease or vascular dementia: a meta-epidemiology study
Source: Front Aging Neurosci. 2023 Apr 26;15:1051809. doi: 10.3389/fnagi.2023.1051809 (PMC10169719; doi:10.3389/fnagi.2023.1051809)
Supplement: Supplementary file 2 [file Table_2.DOC]

**Supplementary Material**

**Table 1~3: Details of the Literature Search Strategy**

(1) PubMed (Aug 16, 2022)

| **Search** | **Query** | **Items found** |
| --- | --- | --- |
| #1 | ("Gout"[Mesh]) | 13478 |
| #2 | Gout*[Title/Abstract] | 17247 |
| #3 | #1OR#2 | 20192 |
| #4 | ("Dementia"[Mesh]) OR "Alzheimer Disease"[Mesh] | 194063 |
| #5 | ((((((Dementia [Title/Abstract]) OR (Alzheimer's disease [Title/Abstract])) OR (Vascular Dementia[Title/Abstract])) OR (Multi-Infarct Dementia[Title/Abstract])) OR (Mixed Dementia[Title/Abstract])) OR (Cognitive decline[Title/Abstract])) OR (Senile Dementia[Title/Abstract]) OR (Cognitive impairment[Title/Abstract])) OR (Cognitive disorder[Title/Abstract])) OR (Cognitive dysfunction[Title/Abstract]))) | 297969 |
| #6 | #4 OR #5 | 346832 |
| #7 | #3 AND #6 | 125 |

(2) Cochrane Library (Aug 16, 2022)

| **Search** | **Query** | **Items found** |
| --- | --- | --- |
| #1 | MeSH descriptor: [Gout] explode all trees | 426 |
| #2 | (gout*):ti,ab,kw | 2018 |
| #3 | #1 OR #2 | 2018 |
| #4 | MeSH descriptor: [Dementia] explode all trees | 6726 |
| #5 | MeSH descriptor: [Alzheimer Disease] explode all trees | 3780 |
| #6 | #4 OR #5 | 6726 |
| #7 | (Dementia): ti,ab,kw OR (Alzheimer's disease):ti,ab,kw OR (Vascular Dementia):ti,ab,kw OR (Multi-Infarct Dementia):ti,ab,kw OR (Mixed Dementia):ti,ab,kw OR (Dementia):ti,ab,kw OR (Alzheimer's disease):ti,ab,kw OR (Vascular Dementia):ti,ab,kw OR (Multi-Infarct Dementia):ti,ab,kw OR (Mixed Dementia):ti,ab,kw | 64461 |
| #7 | #6 OR #7 | 64698 |
| #8 | #3 AND #7 | 24 |

(3) Embase (Aug 16, 2022)

| **Search** | **Query** | **Items found** |
| --- | --- | --- |
| #1 | 'Gout'/exp | 27507 |
| #2 | 'gout*': ti,ab,kw | 24980 |
| #3 | #1 OR #2 | 33188 |
| #4 | 'Dementia'/exp | 415388 |
| #5 | 'Alzheimer Disease'/exp | 229555 |
| #6 | #4 OR #5 | 415388 |
| #7 | 'Dementia':ti,ab,kw OR 'Alzheimers disease':ti,ab,kw OR 'Vascular Dementia':ti,ab,kw OR 'Multi-Infarct Dementia':ti,ab,kw OR 'Mixed Dementia':ti,ab,kw OR 'Dementia':ti,ab,kw OR 'Vascular Dementia':ti,ab,kw OR 'Multi-Infarct Dementia':ti,ab,kw OR 'Mixed Dementia':ti,ab,kw | 193217 |
| #8 | #6 OR #7 | 448005 |
| #9 | #3 AND #8 | 422 |


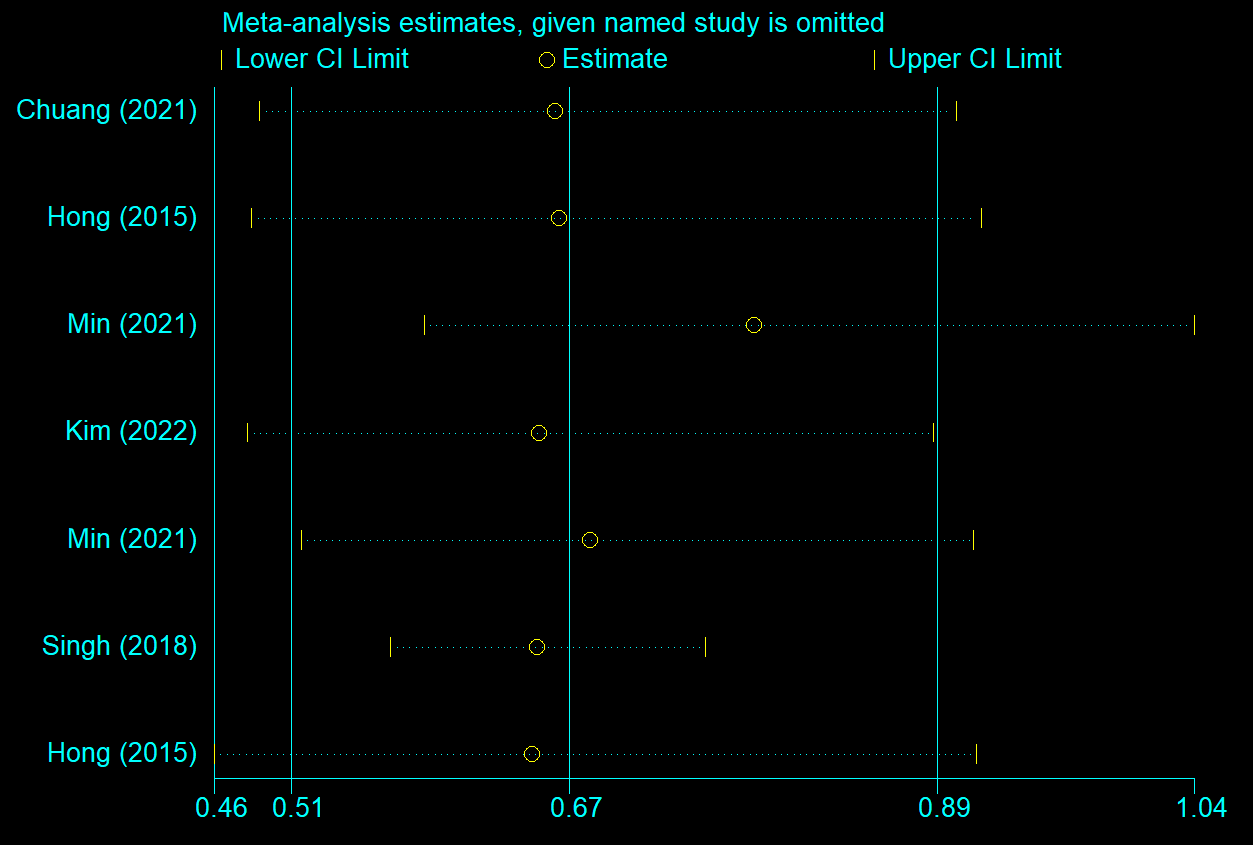


**Figure 1 Sensitivity Analysis of risk of dementia in gout**


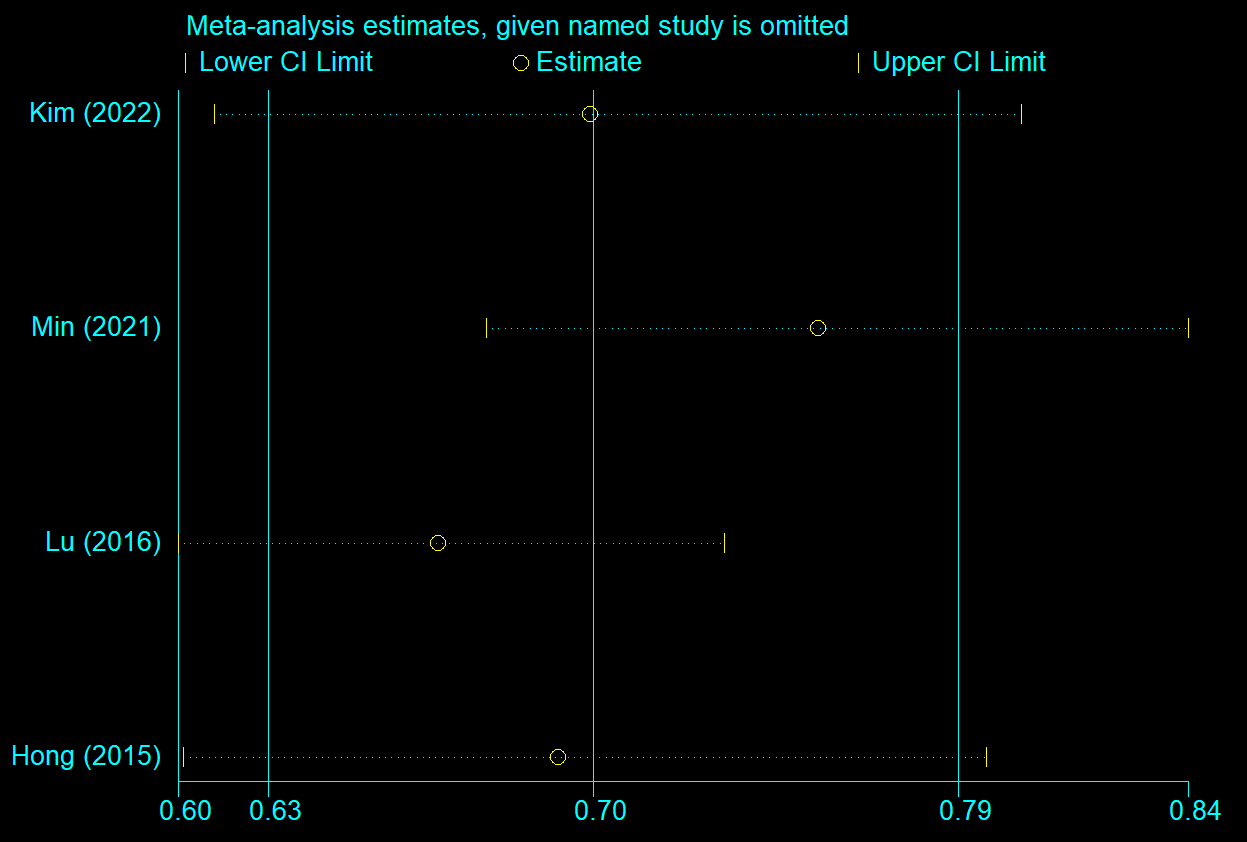


**Figure 2 Sensitivity Analysis of risk of AD in gout**
